# IL-7 Induces an Epitope Masking of *γ*c Protein in IL-7 Receptor Signaling Complex

**DOI:** 10.1155/2017/9096829

**Published:** 2017-01-03

**Authors:** Tae Sik Goh, Yuna Jo, Byunghyuk Lee, Geona Kim, Hyunju Hwang, Eunhee Ko, Seung Wan Kang, Sae-Ock Oh, Sun-Yong Baek, Sik Yoon, Jung Sub Lee, Changwan Hong

**Affiliations:** ^1^Department of Anatomy and Cell Biology, Pusan National University School of Medicine, Yangsan, Republic of Korea; ^2^Department of Orthopedic Surgery, Medical Research Institute, Pusan National University School of Medicine, Busan, Republic of Korea

## Abstract

IL-7 signaling via IL-7R*α* and common *γ*-chain (*γ*c) is necessary for the development and homeostasis of T cells. Although the delicate mechanism in which IL-7R*α* downregulation allows the homeostasis of T cell with limited IL-7 has been well known, the exact mechanism behind the interaction between IL-7R*α* and *γ*c in the absence or presence of IL-7 remains unclear. Additionally, we are still uncertain as to how only IL-7R*α* is separately downregulated by the binding of IL-7 from the IL-7R*α*/*γ*c complex. We demonstrate here that 4G3, TUGm2, and 3E12 epitope masking of *γ*c protein are induced in the presence of IL-7, indicating that the epitope alteration is induced by IL-7 binding to the preassembled receptor core. Moreover, the epitope masking of *γ*c protein is inversely correlated with the expression of IL-7R*α* upon IL-7 binding, implying that the structural alteration of *γ*c might be involved in the regulation of IL-7R*α* expression. The conformational change in *γ*c upon IL-7 binding may contribute not only to forming the functional IL-7 signaling complex but also to optimally regulating the expression of IL-7R*α*.

## 1. Introduction

The common *γ*-chain (*γ*c) cytokines include interleukin-2 (IL-2), IL-4, IL-7, IL-9, IL-15, and IL-21. They have a common feature of sharing *γ*c as the signaling subunit of their receptors [1–3]. Among these cytokines, IL-7 is the pivotal cytokine for the generation of T cells in the thymus and homeostasis in the peripheral lymphoid organs [4–6]. IL-7 binds to IL-7 receptor *α*-chain (IL-7R*α*)/*γ*c, forming functional heterodimeric receptor complex [7]. All these components involved in IL-7 signaling have been implicated in several diseases, such as severe combined immunodeficiency (SCID), autoimmune diseases, and cancers [8–10]. Thus, it is important to understand the molecular mechanism behind the functional IL-7 receptor complex is assembled with IL-7.

There are two proposed models of interaction mechanism between IL-7 and functional receptor complex, which is composed of *γ*c and IL-7R*α*. One is a stepwise cytokine-induced heterodimerization mechanism, in which IL-7 interacts initially with IL-7R*α*, followed by recruitment of *γ*c to form functional signaling complex [7, 11]. Moreover, the other mechanism is through the cytokine-independent preassembled cytokine receptor core [12, 13]. The former has been believed to be more reliable and, thus, conventionally utilized to explain the interaction between cytokines and their receptor core. However, there have been several reports that support the preassembled cytokine receptor core. Since the erythropoietin receptor (EPOR) was first discovered as a preassembled cytokine receptor core, there have been subsequent reports regarding *γ*c cytokine-independent preassembled IL-2R*β*, IL-4R*α*, and IL-9R*α* homodimer or heterodimer [14–17]. The nonfunctional preassembled IL-7R*α*/*γ*c heterodimer and IL-7R*α* homodimer on the cell membrane have been reported [12, 18]. Structural studies suggested that the signal inactive form of unliganded IL-7R*α*/*γ*c heterodimer is converted into the signaling form by a conformational change of the heterodimer in presence of IL-7, denoting that a conformational change in the preassembled IL-7R*α*/*γ*c receptor complex is required for normal signal transduction [12, 13, 19]. However, the mechanism behind conformational change of IL-7R*α* and *γ*c heterotypic receptor has not been fully mapped.

Herein, we show that IL-7 induces epitope masking of *γ*c protein in rapid and energy-independent manner. Anti-*γ*c polyclonal antibody (pAb) detects surface *γ*c protein, while anti-*γ*c monoclonal antibody (mAb) fails to detect the surface *γ*c protein upon IL-7 stimulation. Flow cytometry studies indicate that at least 3 epitopes in the extracellular domain of *γ*c—recognized by 4G3, TUGm2, and 3E12 antibodies—are concealed in the presence of IL-7. We also report that the masking of *γ*c epitopes rapidly arises, and it is IL-7 specific and energy independent. These results reveal that conformational changing sites in *γ*c protein may be located at the 4G3, TUGm2, and 3E12 epitope regions and that it is important to form a functional IL-7R core. Finally, we suggest that these data might support IL-7 signaling model through the IL-7 independent preassembled IL-7R*α*/*γ*c receptor complex.

## 2. Materials and Methods

### 2.1. Animals

C57BL/6 (B6) mice were obtained from the Jackson Laboratory, and *γ*c^−/−^ mice were bred in our own colony. The *γ*c-transgenic construct was made by ligating the *γ*c cDNA into the human CD2 (hCD2) enhancer-promoter-based vector and was injected into the fertilized B6 oocytes to generate *γ*cTg mice [20]. The *γ*c^−/−^ mice were bred with *γ*cTg mice to generate s*γ*c deficient mice. Animal experiments were approved by the Pusan National University Institutional Animal Care and Use Committee (PNU-2014-0620), and all mice were cared for in accordance with the guidelines set forth by the Pusan National University School of Medicine.

### 2.2. Flow Cytometry

The cells were harvested, stained, and analyzed on a FACS Aria or FACS Canto (BD). Dead cells were excluded by forward light scatter gating and propidium iodide staining. Data were analyzed using FlowJo version 10 (TreeStar). Antibodies with the following specificities were used for staining: CD4 (GK1.5 and RM4.5), CD8*α* (53-6-7), TCR*β* (H57-597), *γ*c (4G3, TUGm2 and 3E12), IL-4R*α* (M1), phosphorylated STAT5 (pSTAT5; 47), B220 (30-F11; all from BD), *γ*c (polyclonal Ab; R&D systems), and IL-7R*α* (A7R34; eBiosciences). Anti-human CD3*ε* (Leu4; BD) was used as the isotype control. Anti-mouse CD16/32 antibody (2.4G2; BioLegend) was incubated to block Fc*γ* receptors. All antibodies were incubated at 4°C for 30 min. For FACS analysis of pSTAT5, cells were fixed with 2% paraformaldehyde solution and were subsequently permeabilized with acetone/methanol solution. *y*-axis of all histograms is log scale with maximum value 10^4^. *x*-axis of all histograms is linear scale and maximum values are indicated in each figure legend.

### 2.3. Cell Preparation

A single cell suspension of all LNs from mouse was prepared by applying light pressure with two glass slides to crush the nodes and suspended with chilled full RPMI medium and then stored in ice before using. A single cell of thymus was prepared by tearing into little pieces and suspended with chilled full RPMI medium and then stored in ice before using. The prepared cells were simultaneously stimulated with conditional medium prewarmed at 37°C for the indicated time. The cell viability was comparably maintained during 16-hour incubation regardless of IL-7 treatment (Supplemental Figure 1, left, in Supplementary Material available online at https://doi.org/10.1155/2017/9096829). In case of sodium azide condition, around 20% of cells survived in 16-hour incubation regardless of IL-7 treatment, while cell viability was maintained by 6 hours (Supplemental Figure 1, right). Dead cells were excluded by forward light scatter gating and propidium iodide staining. All FACS analysis was performed only with live cells.

### 2.4. Cell Culture and Cytokine Treatment

Thymocytes and LN cells were cultured in RPMI 1640 medium (Welgene) containing 10% FBS (Gemini), L-glutamine, 100,000 U/ml penicillin plus 100 mg/ml streptomycin (GIBCO), nonessential amino acid (Sigma-Aldrich), sodium pyruvate (Sigma-Aldrich), and *β*-mercaptoethanol (GIBCO). They were stimulated with recombinant human IL-2 (10 ng/ml), murine IL-4 (20 ng/ml), IL-7 (10 ng/ml), IL-9 (100 ng/ml), IL-15 (100 ng/ml), IL-21 (100 ng/ml), or IL-6 (10 ng/ml; all from PeproTech) or stimulated in presence of sodium azide (Sigma-Aldrich) in a 37°C or 4°C humidified incubator.

### 2.5. Immunoblotting

Cell lysates were obtained from IL-7 stimulated T cells and were resolved by SDS-PAGE on 12% acrylamide (Invitrogen) under reducing conditions, which were then transferred to the PVDF membranes (Amersham Biosciences). Blots were incubated with biotin-conjugated anti-*γ*c pAb (R&D system), followed by horseradish peroxidase- (HRP-) conjugated streptavidin (BioLegend).

### 2.6. Quantitative Real-Time PCR

Total RNA was immediately isolated with the Ribospin (GeneAll). RNA was reverse transcribed into the cDNA by oligo(dT) priming with the Reverse Transcription Kit (GeneAll). Quantitative RT-PCR (qRT-PCR) was performed with SYBR Green Master Mix (Bio-Rad) on the LightCycler 96 Real-Time PCR System (Roche) with the following primers: *γ*c (F: 5′-CATGAACCTAGATTCTCCCTGCC-3′; R: 5′-CCAACCAACAGTACACAAAGATCAG-3′), IL-7R*α* (F: 5′-CACACAAGAACAACAATCCCACA-3′; R: 5′-GATCCCATCCTCCTTGATTCTTG-3′), and RPL13 (F: 5′-CGAGGCATGCTGCCCCACAA-3′; R: 5′-AGCAGGGACCACCATCCGCT-3′). Gene expression values were normalized to those of* Rpl13* in the same sample.

### 2.7. Confocal Microscopy

The lymph node (LN) T cells were harvested in RPMI 1640 and stimulated with recombinant human IL-7 (10 ng/ml) at 37°C for the indicated time. After stimulation, cells were washed and fixed with 4% PFA incubation for 10 min. The fixed cells were stained with biotin-conjugated *γ*c-specific monoclonal antibodies (TUGm2: BD) and *γ*c-specific polyclonal antibodies (R&D Systems) at a previously optimized dilution. The Streptavidin Alexa Fluor® 594 was used as a secondary agent. The slides with stained cells were inverted over a drop of vectashield/4,6-diamidino-2-phenylindole (DAPI) and sealed with varnish. Slides were analyzed by confocal microscopy. All images were acquired on an Olympus FV 1000 (Olympus Corp.) using a PLAPON60x/1.35 oil-immersion objective. DAPI fluorescence was detected with a violet 405 nm and Streptavidin Alexa Fluor 594 fluorescence was detected using a violet 543 nm. All confocal images were acquired with frame size of 1024 × 1024 pixels.

### 2.8. Statistical Analysis

Statistical differences were analyzed by unpaired two-tailed Student's *t*-test. *p* values of less than 0.05 were considered significant. ^*∗*^ *p* < 0.05, ^*∗∗*^ *p* < 0.01, and ^*∗∗∗*^ *p* < 0.001. All statistical analyses were performed using GraphPad Prism.

## 3. Results

### 3.1. Detection of Surface *γ*c Protein Using Monoclonal Antibody Is Rapidly Reduced by IL-7

**Figure 1:**
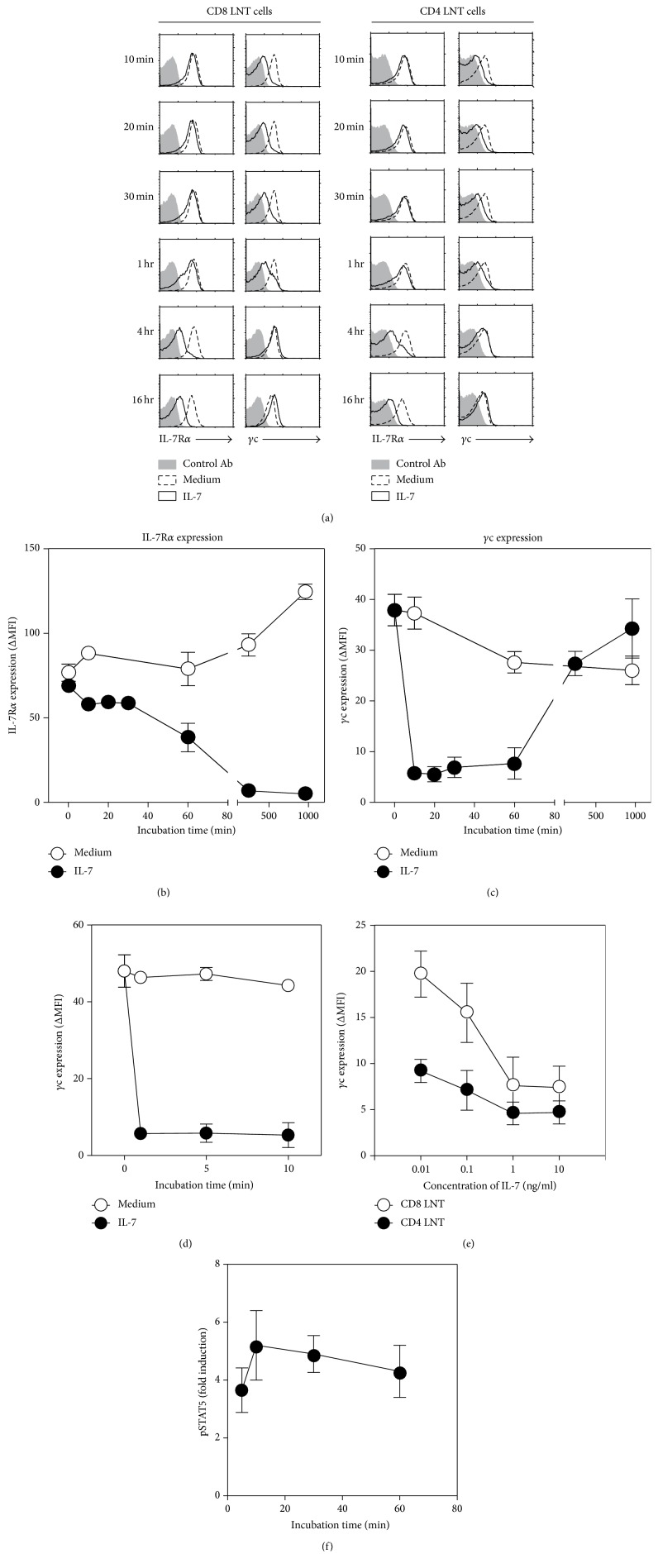
Reduction of *γ*c surface expression with IL-7. (a) Time dependent kinetics of *γ*c expression with IL-7. T cells were stimulated in the presence or absence of IL-7, and the cytokine receptor expression was analyzed by FACS at the indicated time points. The maximum value of *x*-axis is 250 for 10-minute to 4-hour histograms and is 200 for 16-hour histogram. Histograms are representative of six independent experiments. (b, c) Summary of flow cytometry analysis for *γ*c (4G3) and IL-7R*α* expression kinetics in the presence of IL-7. Data are summary of six independent experiments (mean and SEM). (d) The early time point kinetics of *γ*c (4G3) expression in the presence of IL-7. Data are summary of three independent experiments (mean and SEM). (e) Kinetics of *γ*c (4G3) surface expression on T cells under different concentration of IL-7. T cells were stimulated with an indicated concentration of IL-7 for 10 minutes, and the surface *γ*c expression was analyzed by FACS. Data are summary of three independent experiments (mean and SEM). (f) Freshly isolated LNT cells from WT mice were stimulated with IL-7 for the indicated time and assayed for intracellular pSTAT5 content. Fold induction of pSTAT5 content was calculated to that of fresh LNT cells, set as 1. Data are summary of two independent experiments (mean and SEM).

IL-7 downregulates the surface expression of IL-7R*α* with transcriptional mechanism [21]. Since *γ*c is essentially required for normal IL-7 signaling pathway, we wondered how the *γ*c expression is altered in the presence of IL-7 compared with IL-7R*α*. To test this, LN cells were stimulated with IL-7 and analyzed in alteration of *γ*c and IL-7R*α* expression at different time points. Consistent with a previous study [21], IL-7R*α* expression is time-dependently downregulated by IL-7. Surprisingly, surface *γ*c level of CD4^+^ and CD8^+^ LNT cells was rapidly reduced compared with the medium controls, and later their expression was time-dependently rescued, as detected by FACS (Figure 1(a)). It was reversely correlated with the alteration of IL-7R*α* expression pattern upon IL-7 stimulation (Figures 1(b) and 1(c)). Moreover, *γ*c expression on LNT cells disappeared very early after IL-7 stimulation (1 and 5 minutes) (Figure 1(d)), which implies that the alteration in surface *γ*c protein by FACS may not be a physiological phenomenon. Then, we examined the kinetics of surface *γ*c protein under different concentrations of IL-7. This resulted in an IL-7 dependent reduction of surface *γ*c protein on LNT cells (Figure 1(e)). In order to further confirm a coincidence of reduction of *γ*c expression and IL-7 signaling, we have tested kinetics of pSTAT5 induction in early time and found that pattern of pSTAT5 induction was closely correlated with alteration of *γ*c expression (Figure 1(f)). These data further support that reduction of *γ*c expression is dependent on IL-7 binding and signaling.

### 3.2. Reduced Detection of Surface *γ*c Protein Is IL-7 Specific and IL-7R*α* Dependent

**Figure 2:**
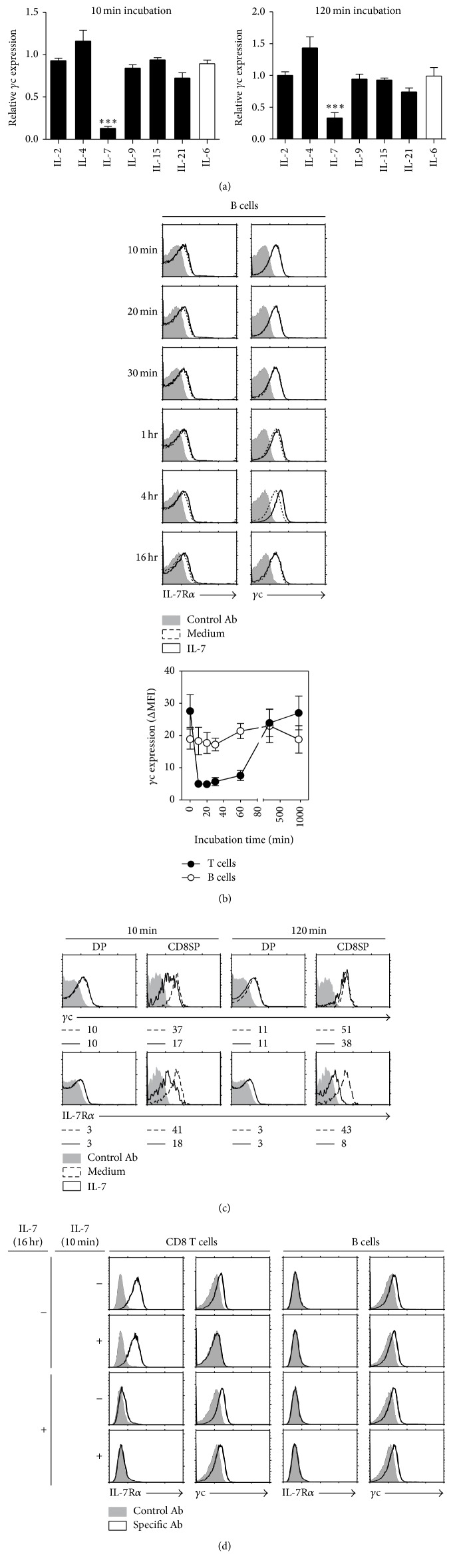
IL-7 specific and IL-7R*α* dependent reduction of *γ*c surface expression. (a) T cells were stimulated with *γ*c cytokines (IL-2, IL-4, IL-7, IL-9, IL-15, and IL-21) or representative non-*γ*c cytokine (IL-6) as negative control for 10 or 120 minutes, and the *γ*c (4G3) expression was analyzed by FACS. The relative *γ*c expression was calculated as the ΔMFI associated with the cytokine-treated group over the ΔMFI associated with the medium control group. Data are summary of more than three independent experiments (mean and SEM). (b) LN cells were stimulated in the presence or absence of IL-7, and the cytokine receptor expression was analyzed by FACS at the indicated time points. The maximum value of *x*-axis is 250 for 10-minute to 4-hour histograms and is 200 for 16-hour histogram. Histograms are representative of five independent experiments. (c) Thymocytes were stimulated in the presence or absence of IL-7, and the *γ*c (4G3) expression was analyzed 10 and 120 minutes after stimulation by FACS. The cells were gated on CD4^+^CD8^+^ and CD4^−^CD8^+^ thymocytes, and the *γ*c (4G3) and IL-7R*α* expression in IL-7 treated cells were compared with IL-7 nontreated thymocytes. The maximum value of *x*-axis is 500 for DP histograms and is 15 for CD8SP histogram. Histograms are representative of three independent experiments. (d) T cells were incubated in the presence or absence of IL-7 for 16 hours, and culture media were changed to fresh media or IL-7 media, and the *γ*c (4G3) expression was analyzed 10 minutes after the media change. The maximum value of *x*-axis is 150 for medium histograms and is 250 for IL-7 pretreated histogram. Histograms are representative of three independent experiments.

*γ*c cytokines, including IL-2, IL-4, IL-7, IL-9, IL-15, and IL-21, share *γ*c as the functional subunit of their signaling. We thus ask whether other *γ*c cytokines change *γ*c surface expression. To address this question, CD8^+^ T cells were stimulated with other *γ*c cytokines and IL-6 as non-*γ*c cytokine control. Upon short-term stimulation (10 minutes), there was a notable and specific decrease in the surface *γ*c protein only for IL-7, whereas no early reduction of surface *γ*c protein was detected for other *γ*c cytokines and IL-6 stimulation (Figure 2(a), upper left). Upon long-term stimulation (120 minutes), although detection of surface *γ*c protein was rescued in IL-7 medium compared to that of short-term stimulation, detection of surface *γ*c protein was still significantly lower than in other *γ*c cytokines and IL-6 medium group (Figure 2(a), upper right). Since mature B cells are negative for IL-7R*α* expression, we stimulated mature B cells with IL-7 to further confirm whether the reduction is dependent on the expression of IL-7R*α*. No reduction of surface *γ*c protein on the mature B cells was detected upon IL-7 stimulation (Figures 2(b) and 2(d)). Moreover, when CD4^+^CD8^+^ (DP) thymocytes, lacking IL-7R*α*, were stimulated with IL-7, there was also no notable reduction of surface *γ*c protein for IL-7 compared with mature CD8^+^ (CD8SP) thymocytes (Figure 2(c)). To offer a greater clarification on whether rapid reduction of surface *γ*c protein is IL-7R*α* dependent, we incubated LN cells with IL-7 or fresh media for 16 hours and stimulated the preincubated LN cells with IL-7 or media for 10 minutes. The *γ*c surface expression of CD8^+^ T cell preincubated in the medium showed a rapid reduction upon IL-7 stimulation whereas IL-7R*α* was expressed in media-stimulated group. However, the rapid reduction of *γ*c surface expression in CD8^+^ T cell preincubated with IL-7 was not observed (Figure 2(d)). Our data demonstrated that only IL-7 could rapidly reduce detection of surface *γ*c protein and the reduction is dependent on IL-7R*α* expression.

### 3.3. Reduced Detection of Surface *γ*c Protein by IL-7 Is Independent of the T Cell's Metabolic Activity

**Figure 3:**
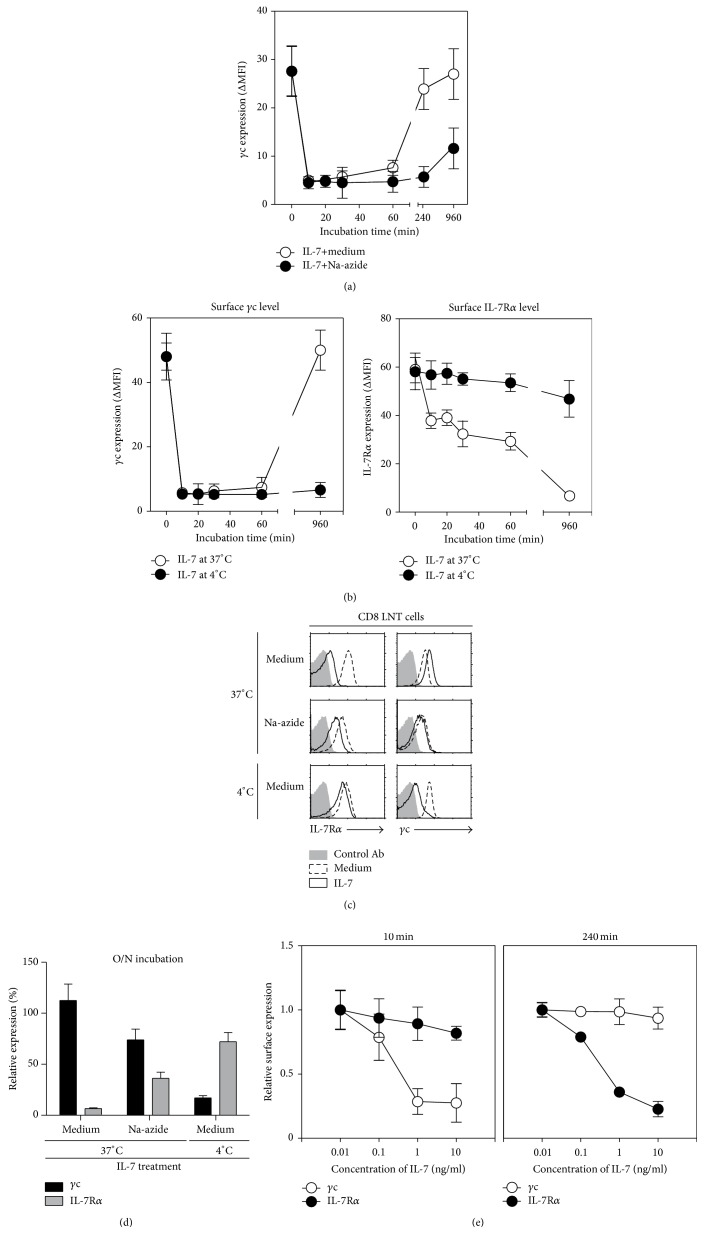
Energy-independent reduction of *γ*c surface expression. (a) T cells were stimulated in the presence of IL-7 plus sodium azide or vehicle, and the *γ*c (4G3) surface expression is analyzed by FACS. Data are summary of three independent experiments (mean and SEM). (b) T cells were stimulated in the presence of IL-7 at 4°C or 37°C, and the *γ*c surface expression is analyzed by FACS. Data are summary of three independent experiments (mean and SEM). (c) T cells were stimulated in the presence of IL-7 plus sodium azide or vehicle at 4°C or 37°C for 16 hours, and the *γ*c (4G3) surface expression is analyzed by FACS. The maximum value of *x*-axis is 200 for 4°C and 37°C medium histograms and is 40 for sodium azide histogram. Histograms are representative of three independent experiments. (d) Relationship between IL-7R*α* downregulation and *γ*c expression. Relative expression was calculated as the ΔMFI associated with IL-7 treated group at the indicated condition over the ΔMFI associated with medium control group at the relevant condition. Data are summary of three independent experiments (mean and SEM). (e) Kinetics of *γ*c (4G3) surface level upon short-term (10 minutes) and long-term (240 minutes) stimulation with different concentration of IL-7. T cells were stimulated with an indicated concentration of IL-7 for 10 and 240 minutes, and the surface *γ*c and IL-7R*α* expression were analyzed by FACS. Data are summary of three independent experiments (mean and SEM).

Since metabolic activity is required for the regulation of surface protein expression [22], we investigated whether the lack of metabolic activity prevents the reduction of *γ*c surface expression. In order to confirm such involvement of metabolic activity in the reduction, CD8^+^ T cells were stimulated with IL-7 in the presence of sodium azide, which acts to block metabolic activity. A reduction of surface *γ*c protein was not prevented by sodium azide (Figure 3(a)). Although surface level of *γ*c protein was partially rescued, 4 hours after the stimulation, it was not perfectly rescued in a sodium azide medium (Figure 3(a)). Furthermore, CD8^+^ T cells stimulated with IL-7 at 4°C also rapidly reduced the surface expression of *γ*c, and it was not subsequently rescued even upon long-term stimulation (16 hours) (Figure 3(b) left). On the other hand, IL-7R*α* expression at 4°C was not significantly changed even under long-term stimulation (Figure 3(b), right), implying that the loss of *γ*c expression was not due to internalization into cells unlike that of IL-7R*α*. We found that *γ*c epitope was immediately concealed in presence of IL-7 and began to be reexposed (60 minutes after stimulation) from when IL-7R*α* expression started to be significantly downregulated, as shown in Figures 1(b) and 1(c). Although IL-7R*α* expression was downregulated at early time (to 30 minutes), IL-7R*α* was still expressed at around 80% compared to medium group. However, *γ*c expression was not detected (around 10%) at the same time points upon IL-7 stimulation. In order to further confirm whether the alteration of *γ*c epitopes is reversely correlated to IL-7R*α* expression, we analyzed pattern of *γ*c and IL-7R*α* expression on different conditions, such as medium, sodium azide, and low temperature (Figures 3(c) and 3(d)). We found that the level of *γ*c surface expression was inversely correlated with the level of IL-7R*α* expression. In addition, we assessed *γ*c and IL-7R*α* expression under both short- and long-term stimulation with different concentration of IL-7. Along with previous experiment, surface *γ*c protein was not detected, while IL-7R*α* expression was slightly downregulated at short time stimulation on concentration dependent manner. Subsequently, the detection of surface *γ*c protein was rescued when IL-7R*α* expression was completely downregulated upon long-term stimulation (Figure 3(e)). Collectively, these results implicate that the alteration of *γ*c epitopes is dependent on surface IL-7R*α* expression and epitope masking of *γ*c is maintained when IL-7R*α* was coexpressed on the cell surface.

### 3.4. IL-7-Induced Epitope Masking of Surface *γ*c Protein

**Figure 4:**
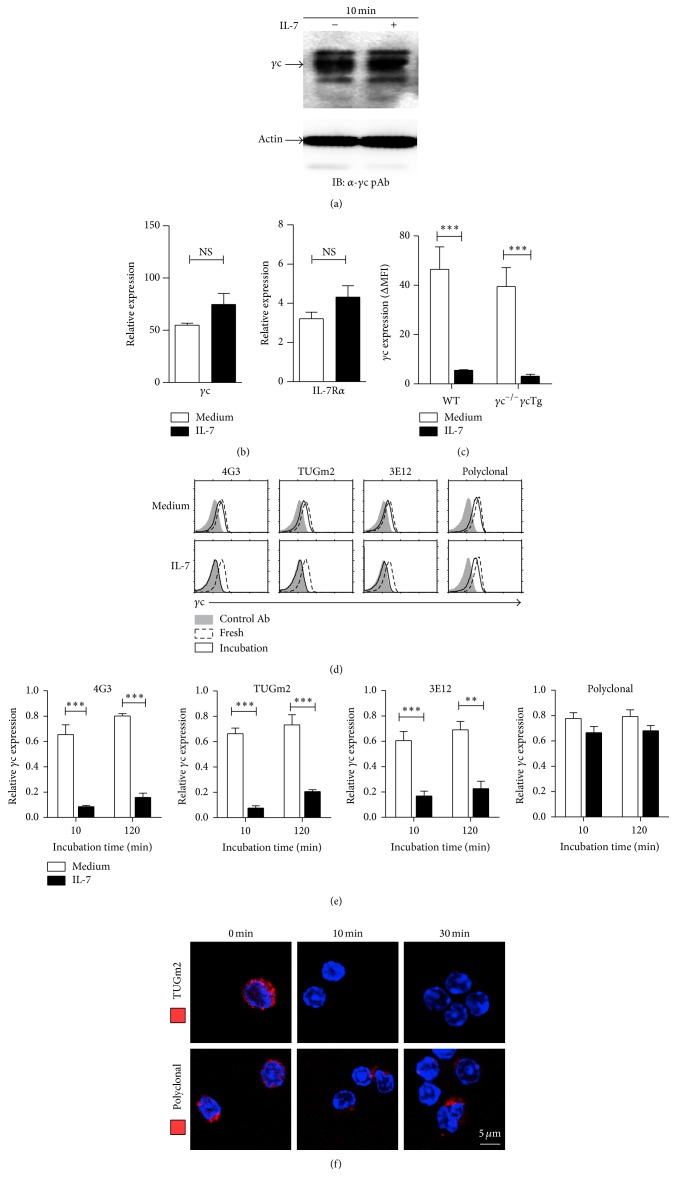
The *γ*c epitope masking by the conformational change of *γ*c. T cells were stimulated in the presence or absence of IL-7 for 10 minutes. (a) *γ*c protein level was analyzed by immunoblot. (b) *γ*c and IL-7Ra mRNA level were measured by real-time PCR. Blots are representative of four independent experiments. Data are summary of four independent experiments (mean and SEM). (c) LNT cells were isolated from WT and *γ*c^−/−^ *γ*cTg mice and stimulated with IL-7 or medium. The *γ*c (4G3) expression was analyzed 10 minutes after stimulation. Data are summary of three independent experiments (mean and SEM). (d) T cells were immunostained with 4G3, TUGm2, 3E12, and anti-*γ*c pAb 10 minutes after IL-7 stimulation. The maximum value of *x*-axis is 250 for all histograms. Histograms are representative of four independent experiments. (e) The relative *γ*c expression was calculated as the ΔMFI associated with T cells incubated at the indicated condition over the ΔMFI associated with fresh T cells. Data are summary of four independent experiments (mean and SEM). (f) Confocal microscopic analysis of surface *γ*c protein. Murine LNT cells were stimulated with IL-7 for 10 and 30 minutes. The fresh or stimulated cells were fixed and stained with biotin-conjugated TUGm2 and pAb. And Streptavidin Alexa Fluor 594 was used to detect surface *γ*c protein (red) and DAPI was used to stain nuclei (blue). Data are representative of three experiments.

Since energy-independent and rapid reduction of *γ*c surface expression was observed only under IL-7 stimulation through a decrease of immunofluorescence analysis by FACS, we assumed that the epitope recognized by 4G3 may be masked on IL-7 binding but may not actually be regulated. To verify this, we first checked the *γ*c protein and mRNA levels of T cells upon either medium or IL-7 stimulation. Both *γ*c protein and mRNA levels were not significantly changed by IL-7 (Figures 4(a) and 4(b)). To further confirm whether the reduction is associated with transcriptional regulation, we used *γ*c^−/−^ *γ*cTg T cells—whereby *γ*c expression is only controlled by human CD2 promoter—to exclude the transcriptional regulation for *γ*c expression. The rapid reduction of surface *γ*c protein was also observed in *γ*c^−/−^ *γ*cTg T cells under IL-7 stimulation (Figure 4(c)). These results imply that the level of *γ*c expression is not regulated by a transcriptional mechanism. We, therefore, used TUGm2 and 3E12 mAb, which recognizes around a unique lysine residue (Lys-158) and a membrane-proximal portion, respectively, to stain T cells under IL-7 stimulation. Interestingly, FACS analysis of different mAb against TUGm2 and 3E12 epitopes has shown similar reduction of *γ*c expression in comparison with 4G3 (Figures 4(d) and 4(e)). Next, pAb for *γ*c was utilized to further confirm whether reduction of surface *γ*c protein is due to the epitope masking of *γ*c protein. The loss of *γ*c expression upon IL-7 stimulation was not observed by pAb after both 10- and 120-minute stimulations, indicating that the level of *γ*c expression is not affected by IL-7 but *γ*c is not detected with the relevant mAb by 4G3, TUGm2, and 3E12 epitope masking (Figures 4(d) and 4(e)). The analysis of confocal microscopy more clearly visualized the epitope masking of surface *γ*c protein by IL-7 in which TUGm2 failed to detect surface *γ*c protein at 10 and 30 minutes, but pAb significantly detected surface *γ*c protein at the same condition (Figure 4(f)). Consequently, these results imply that the epitope masking of *γ*c protein, in which at least 3 epitopes are concealed, is rapidly induced by IL-7 and suggest that IL-7 may bind to the IL-7 preassembled receptor core.

## 4. Discussion

Cytokines are critical for the development and homeostasis of lymphocytes, and they also determine cell fate and differentiation of activated T cells. Among all the cytokines, IL-7 is particularly important, since it is indispensable for the generation of T cells in the thymus and it is critical for T cell homeostasis in the peripheral lymphoid organs [23, 24]. The functional IL-7 receptor consists of the IL-7R*α* chain and the shared *γ*c receptor. Ligand binding leads to a functional receptor core of these two components, inducing the transphosphorylation of receptor-associated kinases JAK1 and JAK3 and activation of STAT5. Genetic deletion of any member of the IL-7R signaling pathway—IL-7R*α*, *γ*c, JAK1, JAK3, and STAT5—results in severely impaired thymopoiesis and T cell function [8, 9]. It has been known that the expression of IL-7R*α* is actively modulated in the management and determination of IL-7 signaling pathway in T cells. Previous studies have shown that IL-7R*α* expression is mainly controlled by a transcriptional regulation under long-term stimulation with IL-7 [21, 25]. In early stage of IL-7 signaling, IL-7 downregulates IL-7R*α* expression by inducing internalization and shifting balance toward lysosome and proteasome-dependent degradation rather than recycling of IL-7R*α* back to the membrane [26, 27]. Under these regulatory mechanisms, IL-7R*α* expression is dynamically regulated, in which IL-7R*α* is upregulated in memory T cells and downregulated in activated and IL-7-primed T cells [21, 25–27]. However, the interaction between *γ*c and IL-7R*α* in all these events has not been explored in detail. Here, we considered it important to address this issue to better understand the biology behind *γ*c cytokines.

**Figure 5:**
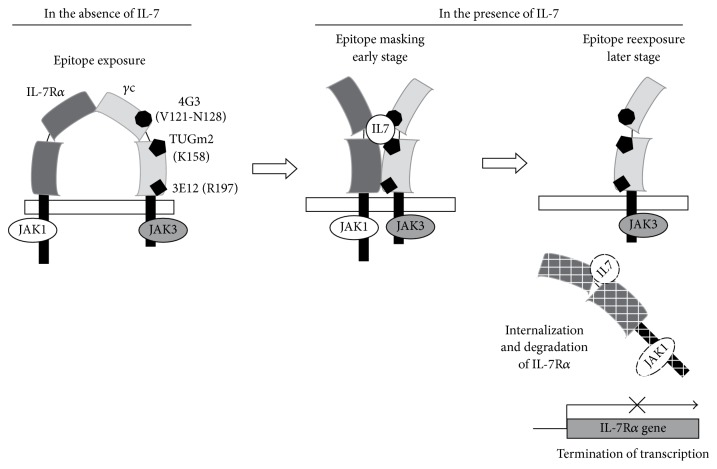
Model of IL-7R*α*/*γ*c receptor core conformational states in the absence or presence of IL-7. This model builds on the concept that binding affinity between IL-7R*α* and *γ*c is present on the cell surface as inactive heterodimers. In the absence of IL-7, ligand independent association of IL-7R*α* and *γ*c would keep JAK1 and JAK3 separated by a large distance to prevent activation. Once IL-7 binds to IL-7R*α*/*γ*c receptor core, the receptors undergo a conformational change, locating the two intracellular domains and activating the signal transduction. At a later stage of IL-7 signaling, IL-7R*α* is internalized, degraded, and transcriptionally terminated, in which the epitopes masked by IL-7/IL-7R*α* are reexposed in free *γ*c.

In this study, we showed that the epitope changes of *γ*c protein, in which at least 3 epitopes are concealed, is rapidly induced by the binding between IL-7 and IL-7 receptor core, and such epitope masking of *γ*c is reversely induced with IL-7R*α* expression. Interestingly, *γ*c epitopes concealed by IL-7 are reexposed with internalization and transcriptional termination of IL-7R*α* (Figure 5). Moreover, the epitope making takes place even at 4°C, indicating its independence of metabolic activity.

IL-7 signaling is mainly controlled by the availability of ligands and the expression of surface cytokine receptors [1]. It is important for T cell homeostasis and responses that their concerted regulation ensures a precisely titrated cytokine response. Thus, it is urgent to expose the genuine molecular mechanism of functioning IL-7R assembly with its relevant ligands.

The sequential receptor dimerization in IL-7 signaling has been thought as the conventional assembly mechanism of IL-7/IL-7R signaling complex [7]. In this conventional model, IL-7 initially interacts with the extracellular ligand binding domain of IL-7R*α*. Once IL-7 and IL-7R*α* are assembled in a one-to-one fashion, *γ*c is engaged with IL-7/IL-7R*α*. This consummated IL-7/IL-7R signaling complex approximates the cytoplasmic tail of both *γ*c and IL-7R*α*, followed by transphosphorylation of JAK kinases to activate several pathways, including the JAK/STAT pathway. Cocrystallization of IL-7 with the extracellular domain of IL-7R*α* in the absence of *γ*c has been reported, supporting the canonical ligand-induced stepwise heterodimerization [28].

Questions still remained regarding the cytokine-induced receptor heterodimerization. For instance, the distances between the C-terminal of mutated IL-7R*α* homodimer in T cell and B cell in acute lymphocytic leukemia (ALL) patients are less than 30 Å, which spontaneously activate the IL-7 signaling pathway to malignant proliferation of lymphocyte in absence of IL-7 [8, 19]. Thus, cytokine-induced receptor heterodimerization may not exactly support the mutated form of IL-7R*α* homodimer detected in both T and B cells in ALL patients [29, 30]. Moreover, several biochemical, biophysical, and structural studies have validated that IL-7R*α* has preassembled mechanism but not sequential dimerization with IL-7 [12, 13, 19]. IL-7 has distinguishing characteristics from other *γ*c cytokines. Compared with other *γ*c cytokines acting on their unique receptor subunit in high affinity, IL-7 interacts with IL-7R*α* in lower binding affinity because its interface has relatively narrow contact area and less polarized and uncharged contact surface and and lacks knobs-into-holes shape complementarity [19]. Unlike other *γ*c cytokine specific receptor subunits that scarcely interact with *γ*c, IL-7R*α* has low binding affinity with *γ*c [19]. Our flow cytometry studies showed that concealing of *γ*c epitope with IL-7 was immediately induced just within 1 minute and even at 4°C at which IL-7R*α* was not downregulated. In keeping with previous reports, these results lead us to consider that recruitment of *γ*c to IL-7/IL-7R*α* which occurs in sequential dimerization model would be impossible in 4°C and sodium azide medium, suggesting that the epitope masking may result from conformational changes of surface *γ*c protein with preassembled heterodimer model [12, 13, 19]. However, we would not exclude the possibility that the ligand-induced stepwise heterodimerization may be involved, because the *γ*c epitopes could be directly concealed by IL-7/IL-7R*α* heterodimer in which 4G3 and TUGm2 epitopes were concealed by IL-7 binding and 3E12 epitope was masked by IL-7R*α*. Along with previous reports, our data may support the preassemble heterodimer model of IL-7R*α*/*γ*c, as shown through the rapid and energy-independent nondetection of surface *γ*c protein.

Conventionally, it has been thought that the different capacity of antibodies in detecting the specific epitopes reflects conformational change [31]. The result of inside-out signaling—increased affinity of integrin—facilitates the denudation of novel epitopes in *α* and *β* subunits located in the extracellular domains [32, 33]. In addition, the conformational change in the associated G(i) subunit of insulin receptor resulted in new epitope exposure in the C-terminal when insulin bound to the insulin receptor [34]. Here, we used 4G3, TUGm2, and 3E12 antibodies, which have been used to define *γ*c cytokine binding motif in *γ*c, and identified that these epitopes are critical for conformational change. Olosz and Malek have reported that 4G3 epitope, which is located near Val-121 and Asn-128 of m*γ*c, is a potential residue of IL-7 binding, although it rarely affects IL-2 and IL-15 binding. The TUGm2 epitope has been predicted to be centered near Lys-158 of m*γ*c, and the antagonistic effect of 4G3 and TUGm2 in the *γ*c cytokine signaling pathway has been demonstrated. Thus, previous studies regarding the cytokine cross-linking assay with mutant molecules, the predicted location of these epitopes, and the inhibitory properties of the mAbs suggest that they are the pivotal sites in direct ligand-receptor interactions [35]. In addition to previous reports, we found that *γ*c surface protein on LNT cells was not detected by immunostaining with 4G3 and TUGm2 mAbs upon IL-7 stimulation, although the expression of *γ*c was intact upon IL-7 stimulation as proved by the immunostaining with anti-*γ*c pAbs, indicating that 4G3 and TUGm2 are essential sites of conformational change as well as ligand binding. Although 3E12 epitope is located in a membrane-proximal portion of m*γ*c, near Arg-197, 3E12 mAb is able to interfere with *γ*c cytokine signaling. Both our research based on the results of flow cytometry assay and previous reports of 3E12 epitope support that 3E12 is a critical site of receptor-*γ*c interaction or conformational change to form the functional receptor core.

Here we report evidence that may support *γ*c cytokine-independent preassembled receptor core mechanism in the IL-7 signaling pathway by flow cytometry evaluation. Walsh et al. assert that this mechanism might be applied to other *γ*c cytokines receptors that form nonfunctioning homodimers and heterodimers [13]. Related to other articles, there would be two or more binding mechanisms of multimeric receptor complex [19, 36]. We believe that the contribution of such binding mechanisms would be different and depends on the ligand species, such as cytokine or hormone. The receptor complex core mechanism in a mainstream of IL-7 signaling pathway needs to be more clarified in further studies.

## 5. Conclusion

In conclusion, our data suggest that IL-7R*α* and *γ*c can spontaneously associate by their extracellular domains on the surface of T cells and form a receptor core, the primary functional target of IL-7. IL-7 binding may induce the conformational change of *γ*c, which migrates to complete the functional receptor core and reconstitutes active protein kinases, such as JAK1-JAK3 pair by clustering their protein components together. Then, the associated JAKs transphosphorylate themselves. The IL-7 signaling pathway induces the internalization and degradation of the surface IL-7R*α*. At this point, epitopes masked by IL-7/IL-7R*α* are reexposed in free *γ*c and are detected with relevant mAbs. Our results thus suggest that the conformational change of *γ*c not only leads the IL-7R core to be functional but also induces the split of IL-7R*α* from *γ*c to be internalized. Controlling the internalization of IL-7R*α* could be one of the alternative strategies to regulate *γ*c responsiveness on IL-7. Further studies are required to further elucidate how *γ*c epitopes are reexposed as IL-7R*α* expression is reduced.

## Supplementary Material

Survival kinetics of WT LN T cells in presence or absent of IL-7 with or without Na-azide. Cell survival was determined by gating on propidium iodide negative cells. Data are summary of three independent experiments (mean and SEM).

## Abbreviations

*γ*c: Common *γ*-chain, CD132
IL: Interleukin
IL-7R*α*: IL-7 receptor *α*-chain
EPOR: Erythropoietin receptor
pAb: Polyclonal Ab
mAb: Monoclonal Ab
FACS: Flow cytometric analysis
LNT cells: Lymph node T cells
JAK: Janus-activated kinase
STAT: Signal transducer and activator of transcription
T-ALL: T cell acute lymphocytic leukemia
B-ALL: B cell acute lymphocytic leukemia
SCID: Severe combined immunodeficiency
TCR: T cell receptor.
