# Supplementary material for: IL-7 Induces an Epitope Masking of γc Protein in IL-7 Receptor Signaling Complex
Source: Mediators Inflamm. 2017 Jan 3;2017:9096829. doi: 10.1155/2017/9096829 (PMC5240581; doi:10.1155/2017/9096829)
Supplement: Supplementary file 1 — Survival kinetics of WT LN T cells in presence or absent of IL-7 with or without Na-azide. Cell survival was determined by gating on propidium iodide negative cells. Data are summary of three independent experiments (mean and SEM). [file 9096829.f1.pdf]

Supplemental figure 1

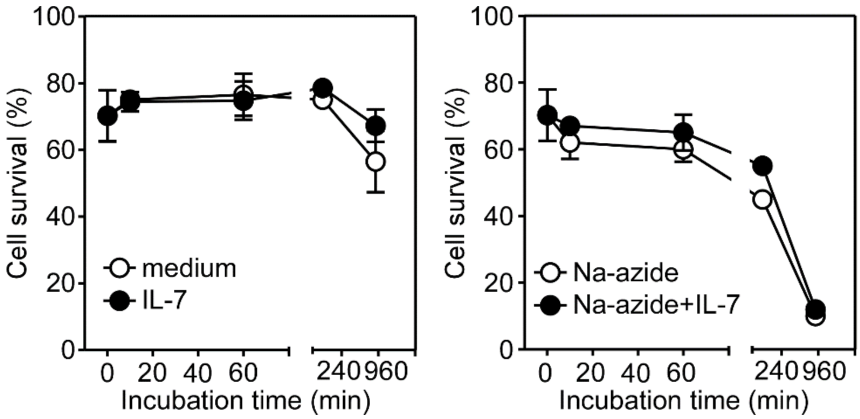

Survival kinetics of WT LN T cells in presence or absent of IL-7 with or without Na-azide. Cell survival was determined by gating on propidium iodide negative cells. Data are summary of three independent experiments (mean and SEM).
